# OD09 How Are Population/Intervention/Comparator/Outcomes Criteria For Pharmaceutical Assessments Determined Around The World?

**DOI:** 10.1017/S0266462324001454

**Published:** 2025-01-07

**Authors:** Skye Newton, Hayley Hill, Tracy Merlin

## Abstract

**Introduction:**

Defining the population, intervention, comparator, outcomes (PICO) criteria is an essential step prior to performing a health technology assessment (HTA), but variations exist in how this step is performed.

**Methods:**

A scoping review was performed to compare the processes and guidance provided for developing the PICO criteria for the assessment of new medicines across Australia, the UK, Canada, the US, European Union (as a single jurisdiction), Germany, France, the Netherlands, South Korea, and Taiwan. The websites of HTA agencies in these jurisdictions were searched for methodological guidance, and PubMed, Embase, and the HTA database were also searched for published literature on the topic of the process or methods for developing the PICO criteria.

**Results:**

Two main approaches are used for developing the PICO criteria. In the UK, US, and European Union, a separate scoping process is used; in the remaining countries, the pharmaceutical manufacturer defines the PICO criteria as part of developing their dossier for submission. Guidance on PICO elements were similar in content but highly varied in the degree of guidance provided. The largest differences were in whether outcomes for people beyond the treated individual were recommended to be assessed.

**Conclusions:**

A separate scoping phase allows stakeholder input into the criteria, which is important with the shift to incorporating more patient input into each phase of HTA. It can come at the cost of timeliness, so requires manufacturers to engage with the HTA systems earlier in the process.

